# Perceptions of HIV and Safe Male Circumcision in High HIV Prevalence Fishing Communities on Lake Victoria, Uganda

**DOI:** 10.1371/journal.pone.0145543

**Published:** 2015-12-21

**Authors:** Paul E. Nevin, James Pfeiffer, Simon P. S. Kibira, Solomon J. Lubinga, Aggrey Mukose, Joseph B. Babigumira

**Affiliations:** 1 Department of Global Health, University of Washington Schools of Medicine and Public Health, Seattle, Washington, United States of America; 2 Health Alliance International, Seattle, Washington, United States of America; 3 Department of Anthropology, University of Washington, Seattle, Washington, United States of America; 4 Department of Community Health and Behavioural Sciences, Makerere University School of Public Health, Kampala, Uganda; 5 Global Medicines Program, Department of Global Health, University of Washington Schools of Medicine and Public Health, Seattle, Washington, United States of America; 6 Pharmaceutical Outcomes Research and Policy Program, University of Washington School of Pharmacy, Seattle, Washington, United States of America; 7 Department of Epidemiology and Biostatistics, Makerere University School of Public Health, Kampala, Uganda; The George Washington University School of Medicine and Health Sciences, UNITED STATES

## Abstract

**Background:**

In 2010, the Uganda Ministry of Health introduced its Safe Male Circumcision (SMC) strategy for HIV prevention with the goal of providing 4.2 million voluntary medical male circumcisions by 2015. Fishing communities, where HIV prevalence is approximately 3–5 times higher than the national average, have been identified as a key population needing targeted HIV prevention services by the National HIV Prevention Strategy. This study aimed to understand perceptions of HIV and identify potential barriers and facilitators to SMC in fishing communities along Lake Victoria.

**Methods:**

We conducted 8 focus group discussions, stratified by sex and age, with 67 purposefully sampled participants in 4 communities in Kalangala District, Uganda.

**Results:**

There was universal knowledge of the availability of SMC services, but males reported high uptake in the community while females indicated that it is low. Improved hygiene, disease prevention, and improved sexual performance and desirability were reported facilitators. Barriers included a perceived increase in SMC recipients’ physiological libido, post-surgical abstinence, lost income during convalescence, and lengthier recovery due to occupational hazards. Both males and females reported concerns about spousal fidelity during post-SMC abstinence. Reported misconceptions and community-held cultural beliefs include fear that foreskins are sold after their removal, the belief that a SMC recipient’s first sexual partner after the procedure should not be his spouse, and the belief that vaginal fluids aid circumcision wound healing.

**Conclusions:**

Previous outreach efforts have effectively reached these remote communities, where availability and health benefits of SMC are widely understood. However, community-specific intervention strategies are needed to address the barriers identified in this study. We recommend the development of targeted counseling, outreach, and communication strategies to address barriers, misconceptions, and community-held beliefs. Interventions should also incorporate female partners into the SMC decision-making process and develop compensation strategies to address lost income during SMC recovery.

## Introduction

Three randomized control trials conducted in Uganda, Kenya, and South Africa have demonstrated that circumcision could reduce heterosexually acquired HIV infection in males by approximately 60% [1–3]. A subsequent post-trial study found that intervention effectiveness is sustained and may reduce HIV acquisition by as much as 73% [4]. These results have important implications for high HIV prevalence countries where heterosexual sexual contact is the primary mode of transmission and circumcision rates are low.

Uganda is one of those countries. According to the 2011 Uganda AIDS Indicator Survey (UAIS), national HIV prevalence in the country had increased from 6.4% to 7.3% since 2005. Additionally, the circumcision rate amongst men aged 15–49 was 26%. The association between circumcision and HIV prevalence is consistent in Uganda, where there is 4.5% prevalence in circumcised men compared to 6.7% in uncircumcised men [5].

In 2010, Uganda’s Ministry of Health followed the recommendations of the World Health Organization (WHO) and Joint United Nations Program on HIV/AIDS (UNAIDS) and introduced its voluntary medical male circumcision strategy, Safe Male Circumcision (SMC). The strategy’s goal is to achieve 80% circumcision in eligible males by providing 4.2 million new safe circumcisions by 2015 [6]. Despite early challenges in intervention scale-up, the SMC campaign is on target, with 1.4 million Ugandan males aged 18+ years having received circumcisions by the end of 2013 [7].

However, according to a September 2014 Uganda AIDS Commission report [8], SMC coverage is not universal. There are several key populations with high HIV prevalence and low access to prevention and treatment services. In Uganda, one key population is fishing communities and the fisher folk who live there. Studies have found fishing communities along Lake Victoria to have HIV prevalence ranging from 22% [9] to 37.1% [7], or approximately three to five times the national average.

There are several unique risk factors that make fishing communities particularly vulnerable to high rates of HIV. Underlying many of the behavioral risk factors found among fisher folk and fishing communities is the mobile and migratory nature of the fishing industry and the consequent absence of social structures in more stable, autochthon communities [10]. Fishing communities are associated with high rates of alcohol consumption, transactional sex, concurrent sexual partnerships, low rates of condom use, and limited knowledge about HIV transmission [11].

Previous qualitative studies have identified pain [12–16], lost income due to missed employment [12,16,17], possible medical complications [13,17], cultural or religious objections [13,17], post procedure abstinence [13,14,17], and fear of HIV testing and results [14,15] as barriers to uptake of voluntary medical male circumcision. Analogously, reported demand facilitators include protection from HIV or other sexually transmitted infections (STIs) [13–17], improved hygiene [13–17], improved sexual desirability and performance [14,16,17], and peer or social influence [13,17]. Female partner support and encouragement is also associated with increased willingness to undergo the procedure [18,19]. However, these studies do not specifically focus on perceptions of SMC within the unique context of rural fishing communities.

Uganda’s 2011–2015 National HIV Prevention Strategy identifies fishing communities as a key affected population that requires “dedicated and targeted comprehensive HIV prevention services tailored to their lifestyle” [20]. Additionally, The Uganda AIDS Commission has indicated that capacity building and identification of effective HIV prevention approaches in key populations is a priority [8]. We therefore conducted this study with the main objective of identifying community perceptions of and popular discourse about HIV and SMC in the unique context of rural Ugandan fishing communities. Specifically, the major aim of our study was to identify facilitators for and barriers to uptake of SMC in the fishing communities of Kalangala District, Uganda.

Kalangala is a district in southern central Uganda comprising of 84 islands on Lake Victoria. The district has an estimated population of approximately 55,000 residents [21]. The local economy is predominantly based on the fishing industry. This paper highlights key study findings that have significant implications for policymakers, project implementers, service providers, and researchers as the SMC campaign continues to be scaled up in Uganda.

## Methods

### Study Design and Recruitment

In May, 2013, we enrolled a total of 67 participants (n = 34 women, n = 33 men) into eight semi-structured focus group discussions (FGDs). We used stratified purposeful sampling to enable the identification of major variations and themes across fairly homogenous strata [22] defined by the characteristics of age (18–24 years or 25+) and sex (male or female). Due to the sensitive and inherently gendered nature of the subject matter, stratifying by sex and age helps mitigate potential intergenerational or gender-based power dynamics that could influence participant responses during FGDs.

We chose to conduct FGDs rather than individual interviews because the aim of the study is to identify community perceptions of HIV and SMC. The use of FGDs allowed us to cast a broader net to capture the diversity of opinions and beliefs that may exist within the community. They are sensitive to cultural variables, enable the elicitation of dominant cultural values, and employ social dynamics that encourage discussion around taboo topics, while allowing participants to explore issues they prioritize, using their own, natural vocabulary and communication methods [23].

The FGDs were conducted in four fishing communities in Kalangala District, Uganda: Bumanji, Kalangala Town Council, Kasenyi, and Mweena. We coordinated recruitment with the District Health Inspector, who directs volunteer Village Health Teams (VHTs) in each of the study communities. VHTs recruited community members according to the sampling strategy predetermined by the study team and coordinated venues for the FGDs.

### Data Collection

All FGDs were conducted in the local language, Luganda, and were facilitated by the same trained moderator and note-taker. The FGDs were semi-structured and based on a prepared FGD guide with 10 general questions about community health, HIV burden, and perceptions of male circumcision. Notes were taken during the FGDs, which were audio recorded and lasted approximately one hour. The FGDs were conducted at diverse sites, including a technical school, a boat landing, boardrooms at the district government headquarters, and bars. We chose these locations because they were deemed to be important locales for community discourse and enabled us to capture maximum variation in community members with diverse socio-demographic factors. Participants were served a refreshment and snack during the discussions.

### Analysis

Audio recordings from each FGD were transcribed and translated to English by native Luganda speakers and imported into *ATLAS*.*ti* software to enable coding and thematic analysis. Prior to coding, we developed a codebook of general themes anticipated during study design. One coder deductively coded from this original codebook while also inductively creating new codes as themes emerged from the data [24]. Major themes were reviewed by on-site, Ugandan members of the research team in order to ensure accuracy, identify discrepancies, and increase validity.

### Ethics

This study was approved by the Makerere University School of Public Health Higher Degrees Research and Ethics Committee and the University of Washington Institutional Review Board. Ethical approval was also granted by the Uganda National Council for Science and Technology. Permission to conduct the study was also granted by Kalangala District officials. We obtained written informed consent from all study participants before the FGDs.

## Results

### Study Participant Characteristics

Our sampling strategy produced a demographically diverse study population spread across the eight FGDs. All participants resided in the four Kalangala District fishing communities (Bumanji, Kalangala Town Council, Kasenyi, and Mweena) where the FGDs were conducted. Participants ranged in age from 18 to 60 years for males and 18 to 50 years for females and had lived in Kalangala for lengths ranging from 4 months to 37 years. See Table 1 for FGD composition details.

**Table 1:** Composition of Focus Groups.

Age and Sex	No. of FGDs	Average No. of Participants/FGD	No. of Participants	Average Participant Age (Years)	Average Length of Residency in Kalangala (Years)
**Younger men (18–24)**	2	8	17	21	3.97
**Older men (25 and above)**	2	8	16	34	11.47
**Younger women (18–24)**	2	9	18	22	7.98
**Older women (25 and above)**	2	8	16	33	12.39
**Overall:**	**8**	**8**	**67**	**27.50**	**8.95**

Education levels for participants ranged from primary level 2 to master’s degree holders. Self-reported participant religion included Pentecostal, Anglican, Catholic, and Muslim. Participant marital status varied across FGDs. Seventy-five percent of older (25+) male and 69% of older female participants reported being married or previously married. Nineteen percent of younger (18–24) males and 44% of younger females identified as married. Participant occupation varied and included fishermen, farmers, students, government employees, and businesspeople.

The following themes of community perceptions and discourse were identified during analysis:

- HIV burden
- Knowledge of and demand for SMC
- Facilitators for SMC
- Barriers for SMC
- Misconceptions about SMC and community-held cultural beliefs

**Fig 1:**
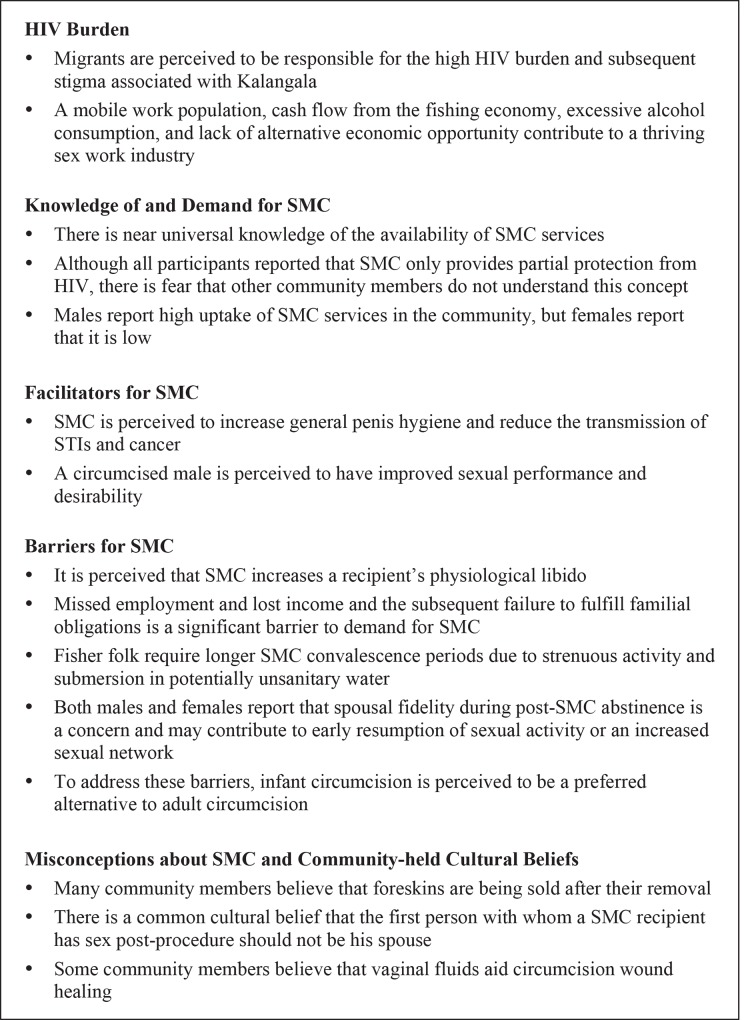
Key Themes and Concepts.

A summary of key themes and concepts identified in the data is presented in Fig 1.

### HIV Burden

In all FGDs, participants acknowledged and expressed concern about the prevalence and burden of HIV in the community. Although several factors were cited as contributing to the problem, the itinerant nature of fishing communities was consistently blamed for the HIV burden. One participant expressed the pervasive sentiment that migrants are bringing disease and contributing to the stigma associated with Kalangala:

*I don’t think that the disease came from nowhere*. *It did not originate from the water*. *No*, *it is the people from the main land who bring the disease*. *The district is affected so much*, *to the extent that Kalangala is known as a place with HIV infected people*.

All FGDs cited the perceived promiscuous lifestyle, migrant work population, and cash flow associated with a fishing economy as major contributors to a thriving sex work industry. One male respondent explained how the influx of fishing money, excessive alcohol consumption, and economic opportunity contribute to the spread of HIV:

*When they* [fishermen] *get these means*, *they go to bars*. *When they get a bit tipsy and their eyes change color*, *even the ugly women appear beautiful to them*. *And women take it as business*, *because they come here when already HIV infected with no other objective but to make money and look for their children’s school fees*, *so they do prostitution*. *They do this prostitution well knowing that they are HIV infected*. *And they work a lot; one woman may sleep with eight men in one night*.

All respondents agreed that condoms were readily available throughout the community, particularly the landing sites, where sex work is concentrated. Despite extensive outreach efforts and the free supply of condoms, there was nearly consensus amongst respondents from both sexes that financial incentives and male dominance in sexual decision-making leads to inconsistent use. One female participant rhetorically asks:

*If you get a man*, *and you have condoms in your house and you tell the man to wear a condom and he declines*, *what do you do*? *Because they give us condoms here for free*. *Should you leave him and walk away*? *I have got myself a man who is going to pay me ten thousand* [Ugandan Shillings (approximately $3–4 USD in 2015)].

Respondents in both male and female FGDs reported that frequently, sex workers and clients will negotiate different prices for protected and unprotected sex. One male participant eloquently connects the practice to the increased risk of acquiring HIV:

*A woman tells you that you should put on a condom and you just say that*, *“I have my money*. *How much do you want*?*” So your money kills you*. *The lady tells you to put on a condom and you refuse*. *Some of us buy the disease ourselves*.

### Community Knowledge of and Demand for SMC

All FGD participants indicated exposure to sensitization efforts and reported knowledge of the existence of SMC services in the community. Additionally, it was universally understood by participants that circumcision only provides partial protection from HIV. In every FGD, participants correctly identified the 60% reduction in transmission of HIV to heterosexual men. However, participants in all four of the older (25+) FGDs also expressed concern that other community members misunderstood the level of protection provided by SMC and that this led to increased risky sexual practices. Only one of the younger (18–24) FGDs conveyed this worry. The perception that SMC is actually increasing the number of HIV infections in the community is exemplified by a response in one of the older male FGDs:

*The people have misunderstood the meaning of SMC*. *People think that they are 100 percent protected and this has increased new infections*.

Despite these concerns, all FGDs agreed that SMC services should be continued in Kalangala.

Although there was universal knowledge of the availability of SMC services, there was variation between male and female FGDs when describing demand and uptake. For example, in one of the male FGDs, a participant noted:

*Very many men have been sharpened …* [the] *majority have been circumcised*.

In contrast, many female FGD participants indicated that despite services being available, uptake was low:

***R1***: *Most of our men are not circumcised; most of them don’t want to be circumcised*, *apart from the Muslims*.

***R2***: *Mine refused because he is a Pentecostal*.

***R3***: *Some of them* [men] *don’t accept it*.

***R4***: *They would rather spend the whole day away than get circumcised*, *yet the people offering the services are usually available*.

### Facilitators for SMC

Participants in all FGDs cited increased protection from HIV and other STIs as an advantage of SMC. Similarly, it was consistently reported that circumcised men are cleaner and more hygienic than those who are uncircumcised. The concept of cleanliness manifests itself in the FGDs in two disparate ways: 1) SMC improves general hygiene of the penis, including the reduction of foul odors. 2) SMC reduces the transmission of STIs via improved cleanliness due to the removal of the foreskin. The latter concept is exemplified by a female participant:

*Most men can spend the entire day without showering*. *Such a man harbors a lot of dirt under the prepuce*, *unlike a circumcised man*. *So that is the source of syphilis*, *HIV and other STIs… it is easy to contract them*, *especially if the woman is not ready for sex*.

In several FGDs, circumcision was described as protective against HIV acquisition because it reduced “bruising.” However, there were inconsistencies with the description of how bruising impacted HIV transmission. When bruising was mentioned in the male FGDs, participants reported that circumcision reduced bruising of the penis and subsequently, risk of HIV transmission. One participant explains:

*A circumcised person can’t easily get HIV*. *‘Asaza kimu’* [he penetrates into the vagina smoothly], *but a person who is not circumcised can easily get infected*. *Since the foreskin keeps on moving up and down*, *he gets bruises and the blood mixes up*, *thus being infected with HIV if the woman is infected*.

However, when discussed in female FGDs, SMC was described as protective against HIV transmission through reduced bruising of the vagina:

*The foreskin keeps a lot of dirt*. *After removing the foreskin*, *the woman does not get bruises so much*. *The bruises are caused by the small size of the woman’s entry point because the skin rubs so much on it if one is not circumcised*.

In several of the FGDs, participants connected SMC with cancer prevention. The mechanisms through which circumcision reduces the risk of cancer were described in varying manners. A male participant explained the relationship between circumcision, pregnancy, and cancer:

*We were taught that the foreskin keeps germs*, *so if you have sex with a pregnant woman before circumcision*, *it may cause cancer*. *One is clean after circumcision to the partner*, *which helps her deliver with no problem*.

Another participant explained that uncircumcised men were less hygienic and therefore more likely to cause cervical cancer via urine:

*In most cases the uncircumcised men cause cervical cancer*, *so circumcision reduces the risk of cervical cancer*. *This is because most men cannot clean this skin thoroughly*, *so whenever he goes to urinate*, *he just shakes the penis and puts it backs*. *The uncleansed urine keeps under the skin and turns into dirt so during sex*, *the skin folds and the dirt goes into the woman*, *thus causing cancer*.

In addition to improved cleanliness and reduced disease transmission, a common theme amongst all FGDs was the improved sexual performance and desirability of circumcised males. The following response from a female participant demonstrates the allure of a circumcised male:

*I have the same point as the former* [participant]. *A circumcised man is more enjoyable during sexual intercourse*. *He doesn’t retire very fast*. *He is better*.

This corroborates the view shared by a male participant that females prefer circumcised males because of prolonged sexual intercourse. He suggests that females may have ulterior motives when encouraging partners to go for SMC:

*No*, *actually it reduces* [sensitivity]. *The science is when they remove the foreskin*, *the sensitivity reduces*, *but the period of performance increases and*, *scientifically*, *ladies would prefer a longer period*. *Removal of the foreskin means that the man takes longer to reach orgasm*, *which comes when you reach full sensitivity*, *so the woman appreciates this long period*. *That is why the woman may encourage you to go for circumcision for hygienic purposes; she will not tell you the truth*.

### Barriers for SMC

All FGDs reported that in addition to improved sexual performance, it is perceived that SMC increases a recipient’s libido. This is perceived to be a physiological change as a result of SMC and is not synonymous with conscious risk compensation due to misconceptions about the level of HIV protection provided. The following response from a female participant typifies the consensus opinion:

*I hear people claiming that after circumcision*, *their libido increases and in that case*, *they are no longer satisfied by one partner or two*, *and just increase the number of partners*.

Loss of income due to missed employment during post-circumcision convalescence was reported by all FGDs to be a significant barrier to demand for SMC. In a fishing economy based on daily earnings, lost employment and income can create household challenges. One male participant explains how it might impact household responsibilities and relationships:

*I think the time you spend on bed rest after circumcision and you cannot do work that requires a lot of energy…*. *Here in Kalangala*, *we are fishermen*. *The time you spend not working*, *you may not have something to eat at home*. *This could even be one of the reasons why you may break up with your partner*.

It was reported that some SMC recipients might be forced to return to work before they have fully healed due to financial constraints. Additionally, one participant clarifies that the fishing profession puts men at higher risk for wound infection and suggests the commonly reported belief that a monetary subsidy to support fishing families is necessary during SMC recovery:

*You fear getting into the water because you may damage the wound*, *so for that period you spend in pain*, *you have nothing to sustain your family*. *At least they should provide some allowance to cater for you*, *even if you need a month*. *One can easily get septic if he goes to fish because we go to deep water for fishing*.

Participants gave varied lengths of time when asked how long an individual should abstain from sex after receiving a circumcision. Responses ranged from two weeks to six months. Some participants explained that individuals heal differently, so they must decide for themselves or listen to the advice of a health worker. Respondents in all FGDs reported that the long period of abstinence following a circumcision was a negative aspect of the procedure. Three of the female FGDs and one of the male FGDs specifically cited the challenge of female spousal fidelity during the healing period. A participant in an older female FGD shared her opinion that the recovery period may lead to domestic conflict or premature resumption of sexual activity:

*If one is to spend two months before having sex*, *I may need to ‘do it’ and I can’t be patient*, *so I go to someone else*. *This may lead to domestic violence or the man may try to have sex so that the wife does not go back for sex with other men*, *so he ends up damaging the wound*.

A participant in an older male FGD offered a similar opinion that SMC actually leads to increased HIV transmission because females are unable to wait for their male counterparts to heal:

*We may think that circumcision is a* [HIV] *prevention method*, *yet it just spreads it*. *This is because the woman knows that it is 40 days to wait and some cannot even be patient for a week*, *so she will go with another man and whatever you wanted to prevent has been destroyed*.

Participants in half of the FGDs suggested that because of the barriers associated with circumcising adult males, campaigns should instead focus on infant circumcision. Three out of these four FGDs went so far as to suggest that circumcision be compulsory for male newborns.

### Misconceptions about SMC and Community-held Cultural Beliefs

Despite several FGDs suggesting financial incentives for SMC, some participants noted that the strategy could have deleterious unintended consequences. One respondent explained how previous compensation strategies have contributed to a pervasive belief among the community that service providers are purchasing and then reselling foreskins:

*In the beginning of SMC*, *they used to offer 30*,*000* [Ugandan shillings (approximately $10 USD in 2015)] *to whoever has been circumcised… you cannot rule it out that the foreskin is not for sale after they have removed it*. *They used to pay money and when more people started going for circumcision*, *they stopped the payment*.

Another male participant offered his opinion on what happens to the foreskin and why this might deter some from receiving SMC:

*Another issue is that these layers that they remove are sold abroad*, *so we say that the government shouldn’t benefit from us so we decide not to… They use them to make women’s creams*.

Half of the FGDs also reported a commonly held belief that a SMC recipient’s first sexual partner post-procedure should not be his wife. This belief was consistent across the sex and age strata of the FGDs. One of the female participants describes the perspective and its potential risks:

*If a man gets circumcised when he has a wife*, *he needs to first sleep with another woman*. *Sleeping with the wife first is a curse*. *This predisposes them to STIs*, *which they may bring back home*.

FGD participants also discussed the belief held by some community members that vaginal fluids promote wound healing and are therefore beneficial for circumcision recovery. There was concern that this practice could increase risk of HIV transmission. The following exchange between participants in an older male FGD illustrates the thought process:

***R1***: *We also have another belief that immediately after circumcision*, *if you have sex*, *the vaginal fluids can help the wound dry quickly*.

***R2***: *Also when you get burned or you get a cut*, *they tell you to go to your wife to take care of the wound*. *So this is the case for circumcision too and the women say*, *‘If you do it gently*, *you will recover faster*.*’*

Another participant noted that the practice is not uncommon:

*If one gets a cut*, *he inserts the finger into the vagina*, *so he may think that it is the case for circumcision*, *so he says let me go and insert the penis*. *This is not childish; people do it*.

## Discussion

SMC services were reported to be widely understood and available Kalangala District, Uganda. However, we identified several barriers to uptake, including: concerns about increased libido, lost income, post-surgical abstinence, and spousal fidelity. We also identified misconceptions about the fate of the foreskin and community-held beliefs regarding post-surgical sexual partners and wound healing that influence demand for and recovery from the procedure. Perceptions of the community’s HIV burden and associated risk factors align with previous studies indicating that Lake Victoria fishing communities have high HIV incidence [25,26] and prevalence [9,27–29], related to high rates of alcohol consumption [25,26,30], and risky sexual behavior [27]. Additionally, the itinerant nature of a mobile fishing population is known to facilitate HIV transmission and impede prevention and treatment [31]. The nebulous cultural and social identity of the fishing communities in Kalangala has enabled an *us and them* attitude that disassociates the community from the “migrants” who are perceived to be one of the main transmitters of HIV. The increased HIV burden and unique risk factors highlight the importance of providing SMC as part of a comprehensive package of HIV prevention interventions in fishing communities.

Current SMC communication and sensitization efforts appear to be reaching a broad audience within the communities. The health benefits of SMC were almost universally reported, and the specific language used by participants indicates exposure to outreach efforts. For example, several community members described reduced “bruising” as the mechanism through which circumcision reduces acquisition of HIV. Bruising is specifically mentioned in several of the SMC campaign materials, as outlined by National Communication Strategy [32]. However, knowledge of the health benefits of SMC alone is not sufficient for demand creation. In a recent study in Rakai, Uganda, only 27% of eligible males were willing to get a circumcision despite the fact that 95% of them understood the health benefits of the procedure [33]. This is consistent with the relatively low demand for services found in this study.

Although sensitization efforts appear to be reaching the community, not all messages were accurately or consistently reported by participants. The varied responses given for length of required abstinence post-circumcision represent a significant barrier to SMC uptake. The same SMC campaign outreach and communication materials that describe reduced “bruising” as a benefit of circumcision clearly state that patients should wait at least six weeks before resuming sexual activity [32]. Accurate knowledge about the length of required post-procedure abstinence is essential for reducing barriers to uptake and potentially negative consequences of the intervention. Required abstinence in sexually active males is a known barrier to uptake of medical male circumcision [13,14,17]. In order to reduce misconceptions, it is important that these sensitization efforts not only reach the community, but that they explicitly describe the mechanisms by which SMC improves health. The disparate responses for how circumcision reduces the risk of cancer and how “bruising” influences disease transmission indicate the need to adapt or improve some components of the SMC communication strategy.

In fishing communities with high rates of risky sexual behavior, as found in Kalangala, fear of partner infidelity or increased risk of HIV acquisition caused by early resumption of sexual activity are a concern. Previous studies in Zambia [34] and Kenya [35] found rates of early resumption of sexual activity after medical male circumcision to be 24% and 31%, respectively. Questions of partner infidelity during post-circumcision recovery were more dominant in female FGDs and indicate that this is not an unwarranted concern amongst men who are eligible for SMC. Female partner support is a known facilitator for undergoing medical male circumcision [18,19] and concerns about partner infidelity have previously been identified as barriers to circumcision uptake [16]. In fishing communities like Kalangala, SMC interventions should take into account sexual concurrency and partner infidelity as potential barriers and should ensure that female partners are incorporated into the circumcision decision-making process. Additionally, the reported endorsement of infant circumcision as a means for addressing these barriers has important implications about the acceptability of the procedure as an HIV prevention strategy.

There are several barriers to SMC uptake that are unique to the fishing profession and reinforce the importance of community-specific intervention efforts. Missed employment and lost income due to circumcision recovery has been identified as a barrier to uptake in other studies on medical male circumcision [12,16,17]. However, this barrier is exacerbated in the fishing communities in Kalangala for two distinct reasons. First, many residents live in poverty and rely on daily fishing income to support themselves and their families. Second, fisher folk may require a longer convalescence period due to the nature of their employment. Fishing is a strenuous activity that requires submersion in potentially unsanitary water. SMC campaign and education materials suggest that patients can return to work after 3 days [36], but there is legitimate concern amongst the community that this may put fisher folk at increased risk for wound infection.

We were able to identify several community perceptions of SMC that act as facilitators for circumcision uptake in Kalangala. Similar to previous studies, we found that improved hygiene [13–17], protection from HIV or other STIs [13–17], and improved sexual desirability and performance [14,16,17] were facilitators for circumcision uptake. Female preference for a circumcised male is highlighted in several SMC campaign materials. A brochure on SMC intended to inform men about the benefits of the procedure states that, “many women also prefer circumcised men for sex because they believe a circumcised penis looks better, is likely to be cleaner, and possibly gives greater sexual satisfaction” [37]. This appears to be reflected in community perception and discourse around SMC in Kalangala.

However, in addition to increased sexual performance, there is a perception in the communities that SMC increases a man’s libido. This change is perceived to be physiological and not only behavioral. Fear of conscious risk compensation has been associated with circumcision as an HIV prevention method since the three randomized control trials first confirmed its protective benefits [38]. The Uganda AIDS Commission specifically lists “disinhibition” as a challenge of the SMC campaign [8]. However, in 2014, the first population-level longitudinal study found no evidence of risk compensation in men who received a medical male circumcision in Kenya [39]. Perceived risk compensation, both physiological and behavioral, is an important factor that influences uptake and acceptability and should be appropriately addressed with outreach and counseling efforts.

Several misconceptions and community-held beliefs about SMC represent unique challenges for scale-up in Kalangala. Although community members suggested a financial subsidy for those seeking SMC, it is possible that previous financial incentives for circumcisions led to a rumor in the community that foreskins were literally being purchased and sold abroad. In a similar situation in Swaziland, male circumcision recipients were incentivized to undergo the procedure with spice packets and promotional items from a spice company. This led to a rumor that foreskins were an ingredient in the spice mixture [40]. It is difficult to know if these misconceptions might be a consequence of misinterpreted incentivization practices or international, Internet-based anti-circumcision campaigns. SMC implementers should target these misconceptions by openly discussing the fate of the foreskin and carefully developing any incentivization schemes.

One community-held belief that impacts SMC uptake is the common perception that after circumcision, a man’s first partner should not be his wife. This cultural belief is not exclusive to Kalangala. In a national supervision report of district level activities, the Uganda AIDS Commission also identified this “misconception” as a challenge to SMC [41]. Policy-makers, implementers, and communication strategists need to develop strategies for confronting these beliefs that increase risky sexual behavior.

Another potentially harmful community-held belief is that vaginal fluids promote wound healing. This belief reportedly leads to early resumption of sexual activity in an effort to expedite healing from circumcision. This puts the circumcision recipient at higher risk of acquiring an STI, especially given other risk factors in the community. SMC implementers should address this practice with proper counseling and sensitization efforts.

### Study Limitations

The exploratory nature of this qualitative study limits the generalizability of our findings. Although we have identified several important factors that influence uptake of SMC in Kalangala that may be relevant in similar contexts, our study is not representative of all rural fishing communities in sub-Saharan Africa. Additionally, individual in-depth interviews with SMC recipients and men who have refused the procedure would allow for more nuanced identification of barriers and facilitators. However, this study was designed to identify the diversity of perceptions and discourse in the community rather than assess influences on individual decision-making. It also would have been beneficial to include key informant interviews with health workers, implementing partners, and community leaders, as they are uniquely knowledgeable about SMC perceptions in the community. This project also does not discuss the anthropological and sociological factors associated with the medicalization of a practice with deep cultural significance. Despite these limitations, our study has generated important evidence for policymakers, project implementers, service providers, and researchers.

## Conclusions

As the SMC campaign continues to scale up in Uganda, it is essential that intervention efforts are tailored to the unique needs and risk factors associated with the country’s key populations. In this study, we have identified several community perceptions of and discourse on HIV and circumcision that influence SMC uptake and acceptability in the fishing communities of Kalangala District. Previous outreach and sensitization efforts have effectively reached the remote communities, where the availability and health benefits of SMC are widely understood. However, intervention strategies must adapt to the specific needs of the community and address some of the barriers we have identified in order to ensure successful implementation. Future research into the individual decision-making process regarding SMC will complement our findings and provide further evidence to improve the link between public health science and practice. We have outlined our recommendations for SMC stakeholders below.

### Recommendations

- Due to the variety of risk factors found in fishing communities, SMC must be offered as part of a comprehensive package of HIV prevention strategies
- SMC service providers and project implementers must account for the specific risk factors for wound healing associated with the fishing profession and carefully address the financial burden of lost employment
- Targeted counseling and outreach are necessary to address the perceived physiological and behavioral changes in libido associated with SMC
- Female partners should be incorporated into the SMC decision-making process to increase uptake and address concerns about post-procedure infidelity
- Specific outreach and communication strategies are needed to address misconceptions and community-held beliefs that influence SMC uptake and potentially increase disease transmission
- Future research is needed to confirm acceptability of infant circumcision as an alternative intervention to adult SMC
